# The prevalence of diabetic retinopathy in type-2 diabetes in Pakistan: a systematic review and meta-analysis

**DOI:** 10.3389/fcdhc.2026.1758759

**Published:** 2026-03-30

**Authors:** Sohail Akhtar, Shafiqa Ashraf, Alessandra Buja, Ibrahim Ahmad Khalil, Olayan Albalawi, Fayaz Ahmad, Muhammad Daud Khan, Aqsa Ali

**Affiliations:** 1 Department of Statistical Science, School of Sciences, University of Padua, Padua, Italy; 2 Department of Mathematics and Statistics, The University of Haripur, Haripur, Pakistan; 3 Department of Cardiological, Thoracic and Vascular Sciences and Public Health, University of Padova, Padova, Italy; 4 School of Public Health, University of Maryland, College Park, MD, United States; 5 Department of Statistics, Faculty of Science, University of Tabuk, Tabuk, Saudi Arabia; 6 Department of Rehabilitation Medicine, The Fifth Affiliated Hospital of Zhengzhou University, Zhengzhou, Henan, China; 7 College of Public Health, Zhengzhou University, Zhengzhou, Henan, China; 8 Department of Public Health, The University of Haripur, Haripur, Pakistan; 9 Department of Statistics, Government College University (GCU) Lahore, Lahore, Pakistan

**Keywords:** diabetes, meta-analysis, Pakistan, public health, retinopathy, systematic review

## Abstract

**Aim:**

Diabetic retinopathy is one the major complications of type-2 diabetes and a leading cause of blindness and visual impairment in Pakistan. However, there is currently no nationally representative population-based estimate of its prevalence. The objective of this study was to determine the pooled prevalence of diabetic retinopathy in individuals with type-2 diabetes in Pakistan.

**Methods:**

We systematically searched MEDLINE (via PubMed), Embase (via Ovid), Web of Science, and local databases from the inception till September 15, 2025. Two independent authors selected studies and conducted the risk of bias analysis. We conducted a random-effects meta-analysis to calculate pooled prevalence. Subgroup analyses and meta-regression analyses were performed in order to explore moderators. Heterogeneity was evaluated incorporating the I² statistic, and robustness of the findings were evaluated from leave-one-out sensitivity analyses. Publication bias was assessed through funnel plots, Begg’s test, and Egger’s test.

**Results:**

In total, 60 studies including 45,646 patients with type-2 diabetes were included in the analysis. The pooled prevalence of diabetic retinopathy was 32.9% (95%CI: 27.67-38.40). Across all studies the 95% prediction interval was wide (2.42–76.28), indicating high level of between-study heterogeneity (I² = 98.8%). There was no evidence of publication bias from Begg’s (p = 0.818) or Egger’s test (p = 0.098). Subgroup analysis by region showed a significant difference (p = 0.0001) in the prevalence of diabetic retinopathy by region, Balochistan 54.65%, Khyber Pakhtunkhwa 47.55%, Punjab 30.60%, Sindh 29.75%, and Islamabad 14.73%. There was a significant time trend (p = 0.034) with increased prevalence of diabetic retinopathy over time, increasing from 21.32% (2001–2009) to 29.31% (2010–2019) and then to 39.59% (2020–2025).

**Conclusions:**

This systematic review and meta-analysis found a high prevalence of diabetic retinopathy in Pakistan with significant heterogeneity among studies. These results substantiate the need for timely screening and management of patients with diabetic retinopathy.

**Systematic review registration:**

https://www.crd.york.ac.uk/PROSPERO/view/CRD42024530689, identifier CRD42024530689.

## Introduction

According to the International Diabetes Federation (IDF), an estimated 537 million adults aged 20 to 79 currently have diabetes, and in the future, this number is expected to increase to 643 million total adults by 2030 and 783 million total adults by 2045 (1). Retinopathy is one of the most common complications impacting those living with diabetes and is a significant contributor to preventable blindness among working-age adults. In fact, it is the fifth leading cause of blindness and moderate-to-severe vision impairment for adults over age 50 in the Global Burden of Disease Study (2). With an ageing world population, people with diabetes living longer, lifestyle factors increasing diabetes-related risks, the growing burden of diabetic retinopathy and demand for eye care and treatment of diabetic retinopathy should increase (3). In many developing countries, basic healthcare services to manage diabetes are frequently non-existent or insufficient and little public awareness or education exists. Poor control of diabetes without diabetes care leads to complications, such as diabetic retinopathy (4, 5). As the prevalence of diabetes continues to rise globally, the number of diabetic retinopathy cases is expected to rise as well (1). Approximately one third of people with diabetes are affected by varying stages of diabetic retinopathy, and diabetic retinopathy is the leading cause of vision impairment and blindness in adults of working-age (6). For both individuals and countries, the economic and social burden of vision loss, which often includes increased dependence on others, loss of income, and increased need for support, is substantial (7, 8).

Diabetes is a major health issue in Pakistan, affecting a large percentage of its population (9–11). As of the year 2021, approximately 26.7% of adults (32.96 million) in Pakistan are estimated to be affected by diabetes (1). Diabetic retinopathy is a serious issue in Pakistan due to diabetes being a leading cause of blindness in the country, and this prevalence is increasing rapidly as a result of urbanization, sedentary lifestyles, and unhealthy eating habits. While a number of studies have reported prevalence rates and other findings on the status of diabetic retinopathy in Pakistan, variations exist between the study findings as a result of methodological differences (for example, sample size and geographic diversity). As a result, it is difficult to make practical generalizations on the national burden and prevalence rate of diabetic retinopathy. It is vital to conduct a systematic review and meta-analysis of diabetic retinopathy in Pakistan to summarize evidence, calculate pooled prevalence rates, and ascertain research gaps. The results will assist policymakers in ensuring healthcare services are suitably allocated and optimal screening, prevention, and interventions are implemented.

## Methods

### Strategy for literature search

The review was preregistered on PROSPERO (CRD42024530689). Following the recommendations of the Preferred Reporting Items for Systematic Reviews and Meta-Analyses (PRISMA) guidelines (12). PRISMA checklist is presented in **Supplementary File 1**.

### Search strategy

A comprehensive review of the literature was undertaken to find articles on diabetic retinopathy in Pakistan. The search strategy included major databases, such as Web of Science, MEDLINE (via PubMed), and Embase (via Ovid), in addition to local Pakistani journals, from inception until September 15, 2025. Furthermore, the reference lists of studies that met the inclusion criteria were scanned for further studies of interest. The search terms were developed using multiple spelling variations, truncated search terms with a wildcard character, and Boolean operators (“AND” and “OR”) to be as inclusive as possible with potentially relevant publications (see **Supplementary File 2**). Two reviewers (A.A. and M.D.K.) conducted the search and study selection independently, and in instances in which there was a conflict, the third reviewer (S.A.) made the final decision.

### Inclusion and exclusion criteria

Studies of potential quantitative synthesis were required to meet the following criteria for inclusion: (i) observational studies reporting on the prevalence of diabetic retinopathy, (ii) studies conducted on a population diagnosed with type-2 diabetes in Pakistan, and (iii) published in English. Excluded studies included: (i) studies that did not provide data to calculate prevalence, (ii) review articles, letters to the editors, theses, (iii) articles that focused on a Pakistani community outside of Pakistan, and (iv) data from multiple studies (only the most recent study was included).

### Assessment of bias risk

Two reviewers (A.A. and I.A.K.) carried out risk of bias assessments utilizing the JBI Critical Appraisal Checklist for Studies Reporting Prevalence Data (13). In line with the checklist, we developed a consistent strategy for using the tool for studies designed to assess diabetic retinopathy. Reviewers assessed each study separately and discrepancies were resolved through discussion or arbitration. A summary quality score was assigned to each study, with all items on the checklist assigned equal weighting. Studies were judged to be high quality (≥70% “Yes” responses), moderate quality (50–69% “Yes” responses), or low quality (≤49% “Yes” responses).

### Data extraction

Related data were independently extracted by two evaluators (I.A.K. and M.D.K.) from each eligible study. The data extracted included: study dates, publication year, first author’s last name, gender, geographic area, median age, sample size, and diabetes duration. If needed, we contacted authors for any missing data. Disagreements were resolved by consensus of the evaluators or with the help of a third evaluator (S.A.).

### Statistical analysis

We used a random-effects approach to obtain a pooled estimate of prevalence of diabetic retinopathy in Pakistan. Additionally, we computed 95% prediction intervals for our summary estimates, to provide a projected range for prevalence estimates in new studies. Between-study heterogeneity was assessed using the I² index (14). Point estimates of diabetic retinopathy were obtained and displayed in forest plots along with the corresponding 95% confidence intervals (CIs). We assessed the influence of individual studies on overall prevalence estimates using sensitivity analysis considering the leave-one-out method (15). In addition, we examined small-study effects by visual assessment with a funnel plot and statistically with Begg’s test (16) and Egger’s regression test (17). We performed subgroup meta-analyses to find out the possible sources of high heterogeneity. Univariable meta-regression models were also used to evaluate the effect of study and participants characteristics by adding covariates. The statistical analysis was conducted using the meta and metafor packages within R statistical software (version 4.5.1). Statistical significance was indicated by a two-sided p-value of less than 0.10. The R² statistic was used to evaluate the degree to which variables in the regression models explain the overall between-study variability. Cohen’s kappa coefficient (κ) was used to determine the degree of consensus among raters regarding study inclusion and data extraction (18).

## Result

**Figure 1:**
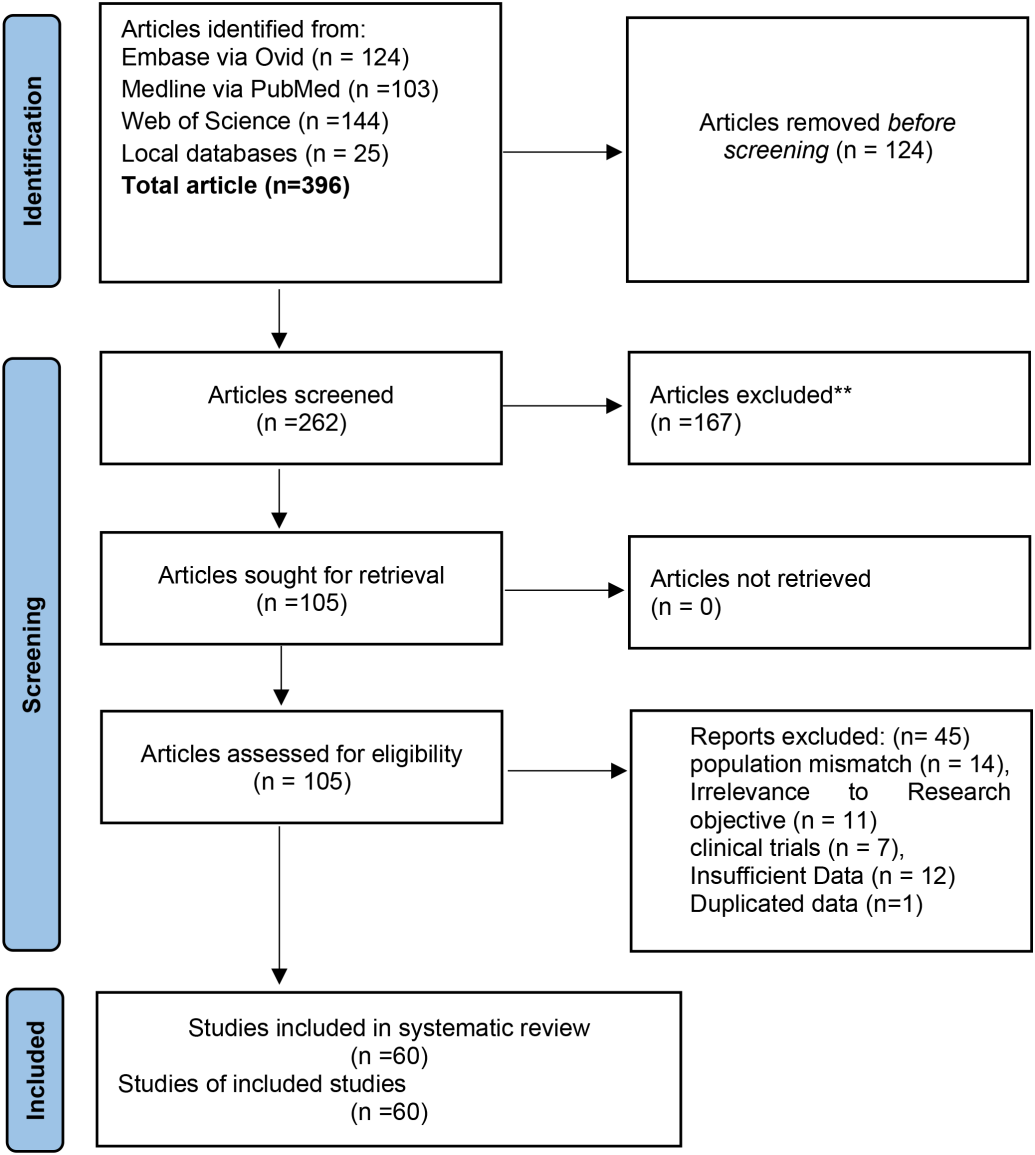
PRISMA flow chart for inclusion and exclusion of studies (13).

A total of 396 articles (**Figure 1**) were identified through electronic databases, including EMBASE (Ovid, n = 124), PubMed (n = 103), Web of Science (n = 144), and local databases (n = 25). After removing 124 duplicate publications through Zotero (version 7) software, 272 unique articles remained for screening. Following the title and abstract review, 167 articles were excluded as irrelevant, and 105 articles were sought for full-text retrieval, all of which were successfully obtained. In the eligibility review, 45 articles were disregarded for the following reasons: population mismatch (n = 14), duplication of dataset (n = 11), outcome not reported (n = 13), ineligible clinical trials (n = 7), and review papers (n = 4). Ultimately, 60 studies satisfied the selection criteria and were included in the qualitative synthesis and quantitative synthesis (meta-analysis).

### Study characteristics

**Supplementary Table 1** (**Supplementary File 3**) provides an overview of 60 studies (19–78) in the analysis. The studies comprised a total of 45646 patients with diabetes, with individual study size varying from 62 (61) to 10,039 (72) participants. The median study size was 220 (IQR, 132 to 634). Age of participants at enrolment was reported in 43 studies, which recruited participants aged 36.95 to 57.4 years (mean (SD), 49 (4. 8)). Geographically, 28 studies (44.9%) were conducted in Punjab, 14 in Sindh, 11 in Khyber Pakhtunkhwa, 2 in the capital city (Islamabad), 1 in Balochistan and 4 across different provinces. The risk of bias assessment showed that 25 studies were rated as low risk, 20 studies as moderate risk, and 15 studies as high risk (**Supplementary File 4**). There was a high level of agreement between authors regarding the extracted data, with a κ value of 0.87 and p-value < 0.001.

### Meta-analysis results

**Figure 2:**
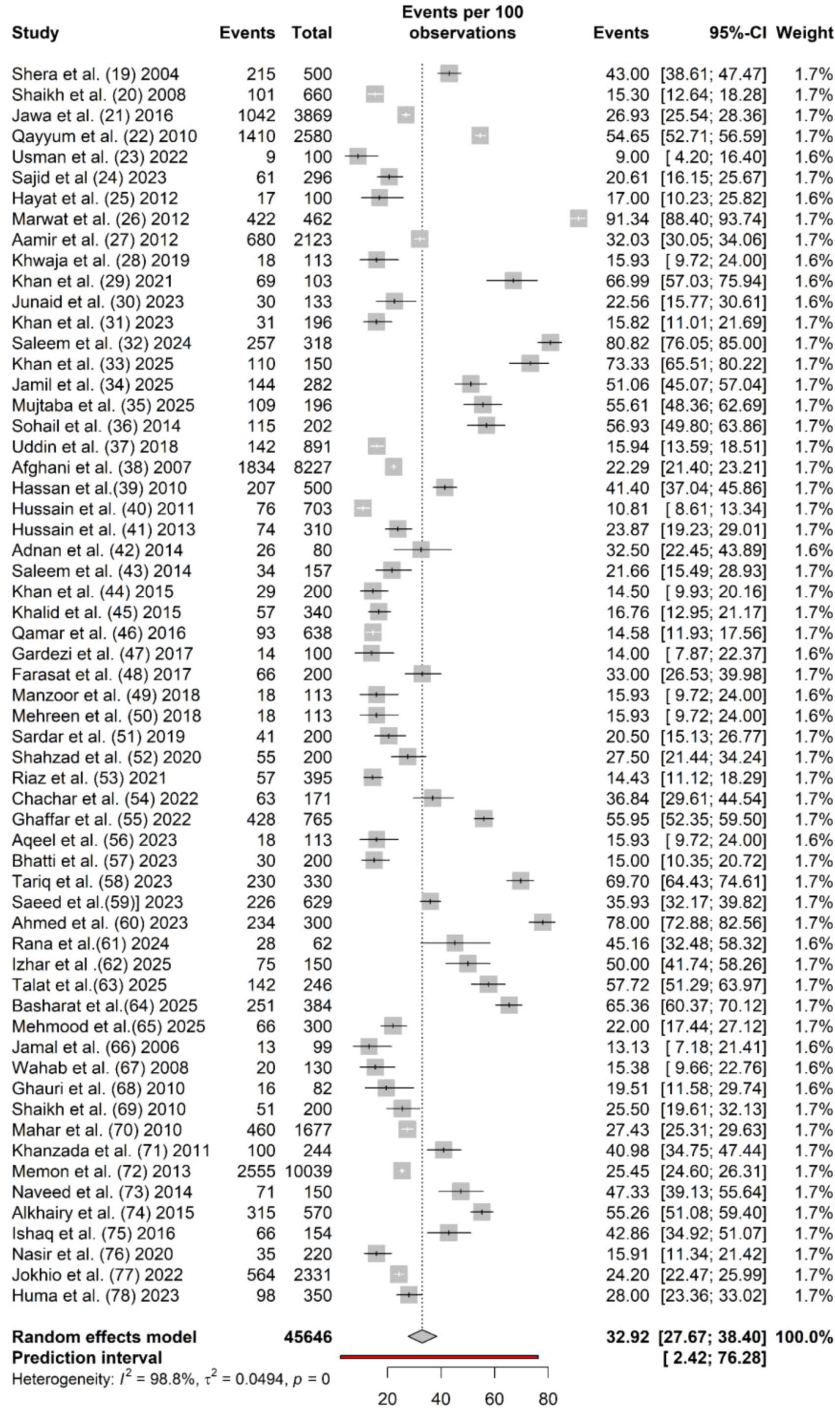
Forest plot for the prevalence of diabetic retinopathy in Pakistan.

**Figure 3:**
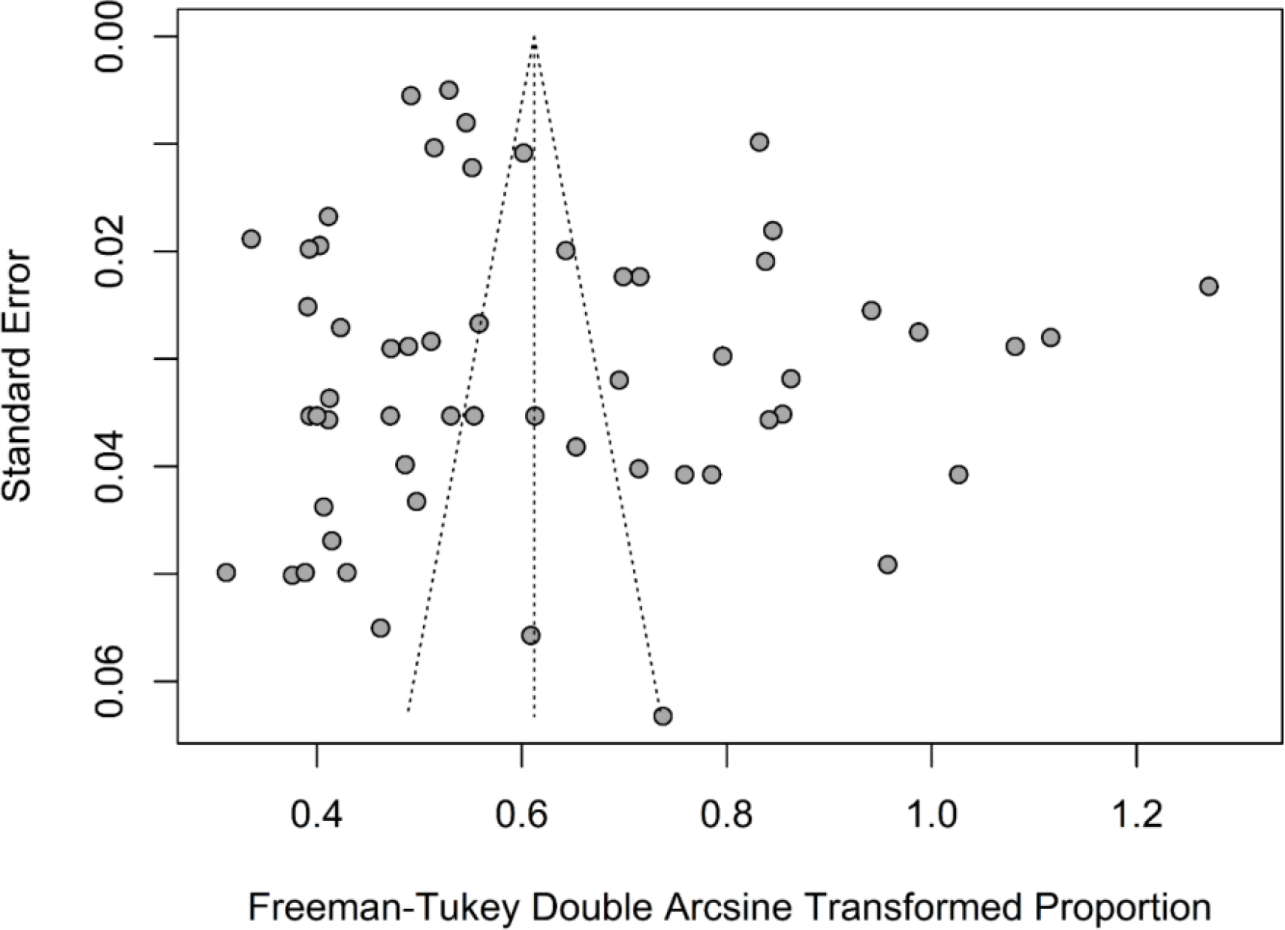
Funnel plot for the prevalence of diabetic retinopathy in Pakistan.

In **Table 1**, there are both overall and subgroup meta-analyses reported for the prevalence of diabetic retinopathy in Pakistan. Among the adult population in Pakistan, prevalence rates of diabetic retinopathy ranged between 9.0% (95% CI: 4.20% 16.40%) and 91.34% (95% CI: 88.40%–93.74%). In **Figure 2**, the prevalence estimates for diabetic retinopathy are shown in a forest plot. The overall pooled estimate based on random-effects analysis from 60 studies indicates a prevalence of 32.92% (95% CI: 27.67%–38.40%). This estimate varied with the 95% prediction intervals ranging between 2.42% and 76.28%. There was a considerable amount of heterogeneity observed across the studies (I² = 98.8%, τ² = 0.049, Q = 4824.13, p <0.001). Review of the funnel plot identified little evidence of publication bias (**Figure 3**), given the nonsignificant Egger test (t = 1.68, df = 58, p-value = 0.0980) and Begg correlation rank test (z = 0.23, p-value = 0.8182).

**Table 1:** Summary statistics overall and subgroup analyses diabetic retinopathy in Pakistan.

Variable	No. of articles	Observations	Events	95% CI	95% prediction interval	I-sq (100%)	P-Value	Begg & Mazumdar	Egger test	Subgroup
Diabetic Retinopathy	60	45646	13836	32.92 (27.67–38.40)	2.42–76.28	98.8	< 0.001	0.8182	0.0980	
By location										0.0001
Balochistan	1	2580	1411	54.65 (52.73–56.57)	-	-				
Khyber Pakhtunkhwa	11	4176	1887	47.55 (29.81–65.61)	0.04–99.33	99.1	< 0.001			
Punjab	28	16126	4490	30.60 (23.66–37.99)	2.17–72.603	98.5	< 0.001			
Sindh	14	16746	4579	29.75 (23.09–36.85)	6.70–60.44	96.6	< 0.001			
Islamabad	2	396	70	14.73 (5.35–27.57)	0.00–53.60	87.1	< 0.001			
By Gender										0.8660
Male	37	10816	3675	32.11 (25.46–39.13)	1.85–76.06	97.1	< 0.001			
Female	36	15861	4469	32.91 (26.12–40.07)	2.10–76.68	97.5	< 0.001			
By time										0.0344
2001-2009	5	9616	2183	21.32 (12.13–32.26)	0.21–61.42	96.9	< 0.001			
2010-2019	29	27110	8233	29.31 (22.82–36.26)	2.23–69.92	98.9	< 0.001			
2020-2025	26	8920	3420	39.59 (30.54–49.01)	2.64–86.49	98.5	< 0.001			
By Mean Age										0.0241
50 years or less	23	7472	2294	27.03 (19.30–35.52)	0.42–72.19	98.7	< 0.001			
Above 50 years	20	11755	4728	41.17 (32.31–50.31)	6.13–82.70	98.7	< 0.001			
Duration of Diabetes										0.0002
Less than 5 years	15	6959	1500	18.54 (12.97–24.80)	1.50–46.97	88.4	< 0.001			
5 Years or Above	14	9007	3743	47.27 (33.26–61.51)	2.13–95.91	99.3	< 0.001			

### Sensitivity analysis

The sensitivity analysis (leave one out) revealed that none of the studies altered the overall pooled prevalence estimate of diabetic retinopathy significantly (**Supplementary File 5**). The prevalence values ranged narrowly between 31.86% - 33.39% with overlapping confidence intervals, compared with the overall random-effects model estimate of 32.92% (95% CI: 27.67%–38.40%). These output findings imply no single study had a disproportionate influence on the summary effect. These findings affirm that no single study exerted undue influence on the summary effect and demonstrate the strength and robustness of the overall meta-analysis estimate in spite of a high level of heterogeneity.

### Subgroup analysis

Subgroup analyses were performed to examine the differences in the prevalence of diabetic retinopathy across different study-level characteristics. When stratified by geographical location, the pooled prevalence varied significantly across regions (P = 0.0001). The highest prevalence was reported in Balochistan (54.65%; 95% CI: 52.73%–56.57%), followed by Khyber Pakhtunkhwa (47.55%; 95% CI: 29.81%–65.61%), Punjab (30.60%; 95% CI: 23.66%–37.99%) and Sindh (29.75; 95% CI: 23.09–36.85). From the 2 studies in Islamabad, the reported prevalence was low at 14.73 (95% CI: 5.35%–27.57%). Gender-based subgroup analysis demonstrated nearly identical prevalence rates for males (32.11%; 95% CI: 25.46%–39.13%) and females (32.91%; 95% CI: 26.12%–40.07%), with no statistically significant differences (p = 0.8660). When stratified by time period, the subgroup analysis revealed a significant increase in prevalence of diabetic retinopathy over time. Between the periods of 2001–2009, prevalence was estimated at 21.32% (95% CI: 12.13%–32.26%), which rose to 29.31% (95% CI: 29.31%–36.26%) in 2010–2019 and further increased to 39.59% (95% CI: 30.54%–49.01%) during 2020–2025. Age-stratified analysis highlighted a significant effect, with individuals younger than 50 years showing a prevalence of 27.03% (95% CI: 19.30%–35.52%), compared to 41.17 (95% CI: 32.31%–50.31%) among those above 50 years (p = 0.0241). One of the major causes of diabetic retinopathy has been the length of staying in the diabetes condition. The pooled prevalence of patients with 5 years or longer diagnosis was 47.27% (95% CI: 33.26%-61.51), and the pooled prevalence of the patients who had a diabetes diagnosis of less than 5 years was 18.54% (95% CI: 12.97%-24.80) (p = 0.0002).

### Meta-regression

These findings of univariate meta-regression analyses (**Table 2**) were that mean age was a significant moderator of the prevalence of diabetic retinopathy (β = 0.0182, 95% CI: 0.0056–0.0319, p = 0.0065, R² = 13.24%), which implied that more prevalence of diabetic retinopathy was reported in studies that involved older people. Although year of publication (β = 0.0092, p = 0.0573, R² = 4.25) and working year (β = 0.0085, p = 0.0540, R² = 4.45) showed positive trends, their effects were marginally nonsignificant. In contrast, sample size (p = 0.5377, R² = 0), study quality (p = 0.1618, R² = 2.05), and female percentage (p = 0.7849, R² = 0) were not significant predictors, indicating negligible influence on prevalence estimates.

**Table 2:** Univariate meta-regression analysis.

Factor	Beta	CI	P	R-Sq (%)
Year	0.0092	-0.0003–0.0188	0.0573	4.25
Sample Size	0	-0.6163–0.5377	0.5377	0
Quality of the studies	0.0652	-0.0261–0.1565	0.1618	2.05
Mean Age	0.0182	0.0056 –0.0319	0.0065	13.24
Working Year	0.0085	-0.0001– 0.0171	0.0540	4.45
Female%	0.0006	-0.0039–0.0052	0.7849	0

## Discussion

This systematic review and meta-analysis provide the most comprehensive evidence to date on the prevalence of diabetic retinopathy among individuals with type-2 diabetes in Pakistan. In the present study, the pooled prevalence of diabetic retinopathy among individuals with type-2 diabetes in Pakistan was 32.92% (95% CI: 27.67%–38.40%). This estimate is considerably higher than those reported in the United Arab Emirates (19.0%) (79), India (21.7%) (80), Mainland China (23.0%) (81), and Peru (23.1%) (82), as well as the global estimate of 25.2% provided by the IDF and the 22.3% reported in a recent worldwide meta-analysis (83). However, our diabetic retinopathy prevalence estimate is lower than the prevalence reported in Puerto Rico (37.7%) (84), and Iran (41.9%) (85). The differences in prevalence between regions may be attributed to variations in study design, country and population characteristics, healthcare infrastructure, and glycemic control. In summary, the results suggest that the burden of diabetic retinopathy in Pakistan is greater than previously reported in most studies from the region and around the world, but it is still below the prevalence of some high-risk populations.

The subgroup analysis of this meta-analysis revealed that the highest prevalence was in Baluchistan (54.65%), followed by Khyber Pakhtunkhwa (47.55%), Punjab (30%), and Sindh (29%). The lowest prevalence was estimated to be in Islamabad (14.73%). These results suggested that the prevalence of diabetic retinopathy varies geographically across Pakistan, which could be attributed to the differences in the management of diabetes, health care access, or other socioeconomic factors that could influence the prevalence. The statistical significance of the differences found in our review (p = 0.01) would suggest that the local-level factors, such as the health system configuration and the screening for diabetic retinopathy, could be playing a role in some of the variations found in our review. In the meta-analysis, we did not find any significant difference in the prevalence with respect to gender, which is in line with the findings of previous studies that found biological sex to have no significant influence on the development of diabetic retinopathy after adjusting for the factors of glycemic control, duration of diabetes, and age (86).

There were also temporal trends, with the prevalence of diabetic retinopathy evidence increasing across the periods of time. For example, the pooled prevalence in 2001–2009 was 21.32%, increasing to 29.31% when looking at 2010–2019, and another increase to 39.59% in 2020–2025 (p = 0.0344). The upward trend could mean there is a true increase in disease burden because of a growing diabetic population, older age, and longer duration of disease, or could also indicate that diagnostic capabilities have improved, and screening utilization has increased through digital retinal imaging technologies, which may have been more recently utilized. We have also seen similar upward trends in other middle-income countries going through an epidemiological transition, which substantiates the worry of similar increases in diabetes complications in Pakistan (87, 88). It reinforces the recommendation of a structured national diabetic retinopathy screening and prevention program, because it appears there is an increase in disease burden. The trend of increased prevalence of diabetic retinopathy that has been observed should be viewed with caution, as it could be due to improved screening and diagnostic practices, and hence should not be considered as a definitive escalation of the disease prevalence. This trend can be better termed as an observed trend rather than a definitive escalation.

Subgroup analyses by age and diabetes duration identified strong relationships that aligned with biological plausibility and prior literature. The prevalence of diabetic retinopathy in participants over the age of 50 years was 41.17%, while the prevalence of retinopathy in people aged 50 years and below was 27.03% (p = 0.0344), reflecting the cumulative effect of aging and prolonged hyperglycemia on the microvasculature. Similarly, patients with diabetes duration greater than five years had a significantly greater prevalence (47.27%) than any group with diabetes for less than five years (18.54%) (p = 0.0002), similar to international studies (89, 90) that consistently identified diabetes duration as the strongest risk factor predicting diabetic retinopathy.

This review has its limitations. Not surprisingly, the studies included in this review had a high heterogeneity, which indicates that around 99% of the observed variability in the estimate of the prevalence of diabetic retinopathy comes from heterogeneity rather than chance. Therefore, the pooled prevalence estimate should be interpreted cautiously as an overall average rather than a precise national estimate, with greater prominence placed on regional provincial estimates and the prediction interval. The observed variability is likely due to differences in criteria for diagnosis, screening procedures, study environment (hospital-based versus community-based), and representation by urban versus rural areas. These factors highlight the importance of context-specific interpretation of the results. We used subgroup analysis and meta-regression models to explore the issue of high heterogeneity. The analysis treated diabetic retinopathy as a single outcome measure without stratification based on severity (such as non-proliferative and proliferative diabetic retinopathy), mainly because of a lack of data at the primary study level. The absence of data related to severity should be noted, as it limits the use of the results for service delivery.

Despite of the limitations we have discussed, this is the first systematic review and meta-analysis to present pooled prevalence of diabetic retinopathy in Pakistan. We published a protocol prior to conducting the study that detailed our methodology and avoided potential duplication. We consider subgroup analysis and random effects meta-regression analysis to account for the multiple factors which may have contributed to our estimate.

## Conclusion

The pooled prevalence of diabetic retinopathy among patients with type 2 diabetes in Pakistan as estimated by this meta-analysis is 32.92%, which is quite high in comparison to the rest of the world. The results highlight the presence of regional differences where the highest prevalence levels occur in Baluchistan and Khyber Pakhtunkhwa and determine age and diabetes duration to be the major risk factors. These analyses demonstrate the necessity of specific screening and management initiatives in order to treat diabetic retinopathy.

## Data Availability

The original contributions presented in the study are included in the article/**Supplementary Material**. Further inquiries can be directed to the corresponding author.
