# Supplementary material for: The prevalence of diabetic retinopathy in type-2 diabetes in Pakistan: a systematic review and meta-analysis
Source: Front Clin Diabetes Healthc. 2026 Mar 30;7:1758759. doi: 10.3389/fcdhc.2026.1758759 (PMC13070823; doi:10.3389/fcdhc.2026.1758759)
Supplement: Supplementary file 2 [file Table2.docx]

**Midline (PubMed) (n=102)**

("Diabetic Retinopathy"[Mesh] OR retinopath*[tiab] OR "diabetic eye disease"[tiab] OR "diabetic retinopathy"[tiab]) AND ("Diabetes Mellitus, Type 2"[Mesh] OR "type 2 diabetes"[tiab] OR "type II diabetes"[tiab] OR T2DM[tiab] OR NIDDM[tiab]) AND (pakistan[ad] OR pakistan[tiab] OR pakistani[tiab])

**Ovid (EMBSE) (n=124)**

1. exp diabetic retinopathy/ OR retinopath*.mp. OR "diabetic eye disease".mp. OR "diabetic retinopathy".mp.2. exp type 2 diabetes mellitus/ OR "type 2 diabetes".mp. OR "type II diabetes".mp. OR T2DM.mp. OR NIDDM.mp. OR "non insulin dependent diabetes".mp.

3. pakistan.mp. OR pakistani.mp. OR (pakistan*).mp.

4. 1 AND 2 AND 3

**Web of Science (n = 124)**

TS=("diabetic retinopathy" OR retinopath* OR "diabetic eye disease" OR "retinal disease*" OR "retinal disorder*" OR "retinal abnormalit*" OR "retinal microvascul*" OR "ocular complication*" OR "vision disorder*" OR "visual impairment") AND TS=("type 2 diabetes" OR "type II diabetes" OR T2DM OR NIDDM OR "adult onset diabetes" OR "non insulin dependent diabetes" OR "diabetes mellitus type 2") AND AD=(Pakistan OR Pakistani)

**Local databases** (Journal of the Pakistan Medical Association, Pakistan Journal of Medical Sciences, etc) (n = 25)
