# Supplementary material for: The prevalence of diabetic retinopathy in type-2 diabetes in Pakistan: a systematic review and meta-analysis
Source: Front Clin Diabetes Healthc. 2026 Mar 30;7:1758759. doi: 10.3389/fcdhc.2026.1758759 (PMC13070823; doi:10.3389/fcdhc.2026.1758759)
Supplement: Supplementary file 3 [file Table3.docx]

| **Table 1: Study Characteristics of all included articles** | | | | | | | | | | |
| --- | --- | --- | --- | --- | --- | --- | --- | --- | --- | --- |
| **Author** | **Year** | **Study Design** | Sample | Positive | **Hospital** | **Province** | **Setting** | Working Year | Mean Age | **Risk of Bais** |
| **Shera et al.** (19) | 2004 | NA | 500 | 215 | yes | Sindh | urban | 2004 | 55.2 | Low |
| **Shaikh et al.** (20) | 2008 | CS | 660 | 101 | no | all | NA | 2003 | NA | Low |
| **Jawa et al.** (21) | 2016 | CS | 3869 | 1042 | no | all | urban | 2015 | NA | Moderate |
| **Qayyum et al.** (22) | 2010 | CS | 2580 | 1410 | yes | Balochistan | urban | 2008 | 51 | Low |
| **Usman et al.** (23) | 2022 | CS | 100 | 9 | yes | Islamabad | urban | 2022 | 47 | Moderate |
| **Sajid et al** (24) | 2023 | CS | 296 | 61 | yes | Islamabad | urban | 2022 | 36.95 | Low |
| **Hayat et al.** (25) | 2012 | CS | 100 | 17 | yes | KP | Urban | 2010 | 45.1 | Moderate |
| **Marwat et al.** (26) | 2012 | CS | 462 | 422 | yes | KP | Urban | 2012 | 48 | Low |
| **Aamir et al.** (27) | 2012 | CS | 2123 | 680 | yes | KP | urban | 2011 | 57.4 | Low |
| **Khwaja et al.** (28) | 2019 | CS | 113 | 18 | yes | KP | Urban | 2018 | 45.46 | Low |
| **Khan et al.** (29) | 2021 | CS | 103 | 69 | yes | KP | urban | 2020 | 54.59 | High |
| **Junaid et al.** (30) | 2023 | CS | 133 | 30 | yes | KP | urban | 2023 | NA | High |
| **Khan et al.** (31) | 2023 | CS | 196 | 31 | yes | KP | urban | 2022 | 37.59 | High |
| **Saleem et al.** (32) | 2024 | CS | 318 | 257 | yes | KP | urban | 2024 | NA | High |
| **Khan et al.** (33) | 2025 | CS | 150 | 110 | yes | KP | urban | 2024 | NA | Low |
| **Jamil et al.** (34) | 2025 | CS | 282 | 144 | yes | KP | urban | 2024 | 56.39 | Low |
| **Mujtaba et al.** (35) | 2025 | CS | 196 | 109 | yes | KP | urban | 2024 | NA | High |
| **Sohail et al.** (36) | 2014 | CS | 202 | 115 | no | all | NA | 2010 | 52.9 | Low |
| **Uddin et al.** (37) | 2018 | CS | 891 | 142 | no | all | URBAN | 2016 | 47.7 | Low |
| **Afghani et al.** (38) | 2007 | CS | 8227 | 1834 | both | Punjab | both | 2001 | NA | Low |
| **Hassan et al.**(39) | 2010 | NA | 500 | 207 | yes | Punjab | urban | 2005 | NA | Moderate |
| **Hussain et al.** (40) | 2011 | CS | 703 | 76 | yes | Punjab | urban | 2009 | NA | Low |
| **Hussain et al.** (41) | 2013 | CS | 310 | 74 | yes | Punjab | urban | 2012 | 49.04 | Low |
| **Adnan et al.** (42) | 2014 | CS | 80 | 26 | yes | Punjab | urban | 2012 | 51 | Moderate |
| **Saleem et al.** (43) | 2014 | CS | 157 | 34 | yes | Punjab | urban | 2014 | 41.65 | High |
| **Khan et al.** (44) | 2015 | CS | 200 | 29 | yes | Punjab | urban | 2012 | 51.05 | Moderate |
| **Khalid et al.** (45) | 2015 | CS | 340 | 57 | yes | Punjab | urban | 2015 | 47.55 | Low |
| **Qamar et al.** (46) | 2016 | CS | 638 | 93 | no | Punjab | urban | 2015 | 45.75 | Low |
| **Gardezi et al.** (47) | 2017 | NA | 100 | 14 | yes | Punjab | urban | 2016 | 45.31 | Higj |
| **Farasat et al.** (48) | 2017 | CS | 200 | 66 | yes | Punjab | urban | 2010 | 50.77 | High |
| **Manzoor et al.** (49) | 2018 | CS | 113 | 18 | yes | Punjab | urban | 2017 | 45.46 | Moderate |
| **Mehreen et al.** (50) | 2018 | NA | 113 | 18 | yes | Punjab | urban | 2017 | 45.4 | Moderate |
| **Sardar et al.** (51) | 2019 | NA | 200 | 41 | yes | Punjab | urban | 2018 | 46.5 | Moderate |
| **Shahzad et al.** (52) | 2020 | CS | 200 | 55 | yes | Punjab | urban | 2019 | NA | High |
| **Riaz et al.** (53) | 2021 | CS | 395 | 57 | yes | Punjab | urban | 2019 | 52.93 | Moderate |
| **Chachar et al.** (54) | 2022 | CS | 171 | 63 | yes | Punjab | urban | 2021 | 51.1 | High |
| **Ghaffar et al.** (55) | 2022 | CS | 765 | 428 | yes | Punjab | urban | 2021 | 42.18 | Moderate |
| **Aqeel et al.** (56) | 2023 | CS | 113 | 18 | yes | Punjab | urban | 2022 | 45.4 | High |
| **Bhatti et al.** (57) | 2023 | NA | 200 | 30 | yes | Punjab | urban | 2022 | 51.5 | High |
| **Tariq et al.** (58) | 2023 | CS | 330 | 230 | yes | Punjab | urban | 2018 | 50.4 | Moderate |
| **Saeed et al.**(59)**]** | 2023 | CS | 629 | 226 | yes | Punjab | both | 2023 | NA | Moderate |
| **Ahmed et al.** (60) | 2023 | CS | 300 | 234 | yes | Punjab | urban | 2023 | 54.76 | Moderate |
| **Rana et al.**(61) | 2024 | CS | 62 | 28 | yes | Punjab | urban | 2022 | 49.37 | High |
| **Izhar et al .**(62) | 2025 | CS | 150 | 75 | yes | Punjab | urban | 2024 | 48.9 | Low |
| **Talat et al.**(63) | 2025 | CS | 246 | 142 | yes | Punjab | urban | 2024 | 49.26 | Low |
| **Basharat et al.**(64) | 2025 | CS | 384 | 251 | yes | Punjab | urban | 2024 | 54.96 | Moderate |
| **Mehmood et al.**(65) | 2025 | CS | 300 | 66 | yes | Punjab | urban | 2023 | 52 | Low |
| **Jamal et al.** (66) | 2006 | NA | 99 | 13 | no | Sindh | urban | 2002 | NA | High |
| **Wahab et al.** (67) | 2008 | CS | 130 | 20 | yes | Sindh | urban | 2006 | 43.2 | Low |
| **Ghauri et al.** (68) | 2010 | CS | 82 | 16 | yes | Sindh | urban | 2009 | NA | Moderate |
| **Shaikh et al.** (69) | 2010 | NA | 200 | 51 | yes | Sindh | urban | 2010 | 49 | Low |
| **Mahar et al.** (70) | 2010 | CS | 1677 | 460 | no | Sindh | urban | 2008 | 42 | Low |
| **Khanzada et al.** (71) | 2011 | CS | 244 | 100 | yes | Sindh | urban | 2010 | NA | Low |
| **Memon et al.** (72) | 2013 | CS | 10039 | 2555 | no | Sindh | urban | 2011 | NA | Low |
| **Naveed et al.** (73) | 2014 | CS | 150 | 71 | yes | Sindh | urban | 2012 | NA | High |
| **Alkhairy et al.** (74) | 2015 | CS | 570 | 315 | yes | Sindh | urban | 2012 | 52.3 | Moderate |
| **Ishaq et al.** (75) | 2016 | CS | 154 | 66 | yes | Sindh | urban | 2014 | 50.59 | Moderate |
| **Nasir et al.** (76) | 2020 | CS | 220 | 35 | yes | Sindh | urban | 2016 | NA | Moderate |
| **Jokhio et al.** (77) | 2022 | CS | 2331 | 564 | no | Sindh | rural | 2018 | 54.93 | Low |
| **Huma et al.** (78) | 2023 | CS | 350 | 98 | yes | Sindh | urban | 2021 | 56.1 | Moderate |
