# Supplementary material for: The prevalence of diabetic retinopathy in type-2 diabetes in Pakistan: a systematic review and meta-analysis
Source: Front Clin Diabetes Healthc. 2026 Mar 30;7:1758759. doi: 10.3389/fcdhc.2026.1758759 (PMC13070823; doi:10.3389/fcdhc.2026.1758759)
Supplement: Supplementary file 4 [file Table4.docx]

|  | | | | | | | | | | | |
| --- | --- | --- | --- | --- | --- | --- | --- | --- | --- | --- | --- |
| Table S1: **Study Characteristics of all included articles** | | | | | | | | | | | |
| **Author** | **Year** | 1. Was the sample frame appropriate to address the target population? | 2. Were study participants sampled in an appropriate way? | 3. Was the sample size adequate? | 4. Were the study subjects and the setting described in detail? | 5. Was the data analysis conducted with sufficient coverage of the identified sample? | 6. Were valid methods used for the identification of the condition? | 7. Was the condition measured in a standard, reliable way for all participants? | 8. Was there appropriate statistical analysis? | 9. Was the response rate adequate, and if not, was the low response rate managed appropriately? | Quality |
| **Shera et al.** (19) | 2004 | 1 | 1 | 1 | 1 | 1 | 1 | partial | 1 | 1 | Low |
| **Shaikh et al.** (20) | 2008 | 1 | 1 | 1 | 1 | 1 | 1 | 1 | 1 | 1 | Low |
| **Jawa et al.** (21) | 2016 | 1 | partial | 1 | 1 | 1 | partail | partial | 1 | 1 | Moderate |
| **Qayyum et al.** (22) | 2010 | 1 | 1 | 1 | 1 | 1 | 1 | 1 | partial | 0 | Low |
| **Usman et al.** (23) | 2022 | partial | partial | Moderate | 1 | 1 | 1 | 1 | partial | 1 | Moderate |
| **Sajid et al** (24) | 2023 |  |  |  |  |  |  |  |  |  | Low |
| **Hayat et al.** (25) | 2012 | 1 | 1 | partial | 1 | 1 | partail | partial | 1 | 1 | Moderate |
| **Marwat et al.** (26) | 2012 | 1 | 1 | 1 | 1 | 1 | 1 | 1 | 0 | unclear | Low |
| **Aamir et al.** (27) | 2012 | 1 | 1 | 1 | 1 | 1 | 1 | 1 | 1 | 0 | Low |
| **Khwaja et al.** (28) | 2019 | 1 | 1 | partial | 1 | 1 | 1 | 1 | 1 | 0 | Low |
| **Khan et al.** (29) | 2021 | partial | 0 | partial | 1 | 1 | 1 | partial | 1 | 0 | High |
| **Junaid et al.** (30) | 2023 | partial | partial | 1 | 1 | 1 | partial | partial | 1 | 0 | High |
| **Khan et al.** (31) | 2023 | 1 | partial | partial | 1 | 1 | partial | partial | 1 | 0 | High |
| **Saleem et al.** (32) | 2024 | partial | 0 | 1 | 1 | 1 | partial | partial | 1 | 0 | High |
| **Khan et al.**(33) | 2025 | 1 | 1 | 1 | 1 | 1 | 1 | 1 | 1 | 0 | Low |
| **Jamil et al.**(34) | 2025 | 1 | partial | 1 | 1 | 1 | 1 | 1 | 1 | 0 | Low |
| **Mujtaba et al.**(35) | 2025 | 0 | 0 | 1 | 1 | 1 | unclear | unclear | 1 | unclear | High |
| **Sohail et al.** (36) | 2014 | 1 | 1 | 1 | 1 | 1 | 0 | 1 | 1 | 1 | Low |
| **Uddin et al.** (37) | 2018 | 1 | 1 | 1 | 1 | 1 | 1 | 1 | 1 | 1 | Low |
| **Afghani et al.** (38) | 2007 | 1 | 1 | 1 | 1 | 1 | 0 | 1 | 1 | unclear | Low |
| **Hassan et al.**(39) | 2010 | 1 | partial | 1 | 1 | 1 | partial | partial | 1 | 1 | Moderate |
| **Hussain et al.** (40) | 2011 | 1 | 1 | 1 | 1 | 1 | 1 | 1 | 1 | 0 | Low |
| **Hussain et al.** (41) | 2013 | 1 | partial | 1 | 1 | 1 | 1 | 1 | 1 | 0 | Low |
| **Adnan et al.** (42) | 2014 | 1 | 0 | 1 | 1 | 1 | 1 | partial | 1 | 0 | Moderate |
| **Saleem et al.** (43) | 2014 | 1 | 0 | partial | 1 | 1 | partial | partial | 1 | 0 | High |
| **Khan et al.** (44) | 2015 | 0 | 1 | 1 | 1 | 1 | 0 | 1 | 0 | unclear | Moderate |
| **Khalid et al.** (45) | 2015 | 1 | 1 | 1 | 1 | 1 | 1 | 1 | 1 | 0 | Low |
| **Qamar et al.** (46) | 2016 | 0 | 1 | 1 | 1 | 1 | 1 | 1 | 1 | unclear | Low |
| **Gardezi et al.** (47) | 2017 | 1 | partial | partial | 1 | 1 | partial | partial | 1 | 0 | High |
| **Farasat et al.** (48) | 2017 | 1 | partial | partial | 1 | 1 | partial | partial | 1 | 0 | High |
| **Manzoor et al.** (49) | 2018 | 1 | 1 | partial | 1 | 1 | 1 | partial | 1 | 0 | Moderate |
| **Mehreen et al.** (50) | 2018 | 1 | 1 | partial | 1 | 1 | 1 | partial | 1 | 0 | Moderate |
| **Sardar et al.** (51) | 2019 | 1 | partial | 1 | 1 | 1 | 1 | partial | 1 | 0 | Moderate |
| **Shahzad et al.** (52) | 2020 | partial | 0 | partial | 1 | 1 | partial | partial | 1 | 0 | High |
| **Riaz et al.** (53) | 2021 | 1 | partial | 1 | 1 | 1 | 1 | partial | 1 | 0 | Moderate |
| **Chachar et al.** (54) | 2022 | 1 | partial | partial | 1 | 1 | partial | partial | 1 | 0 | High |
| **Ghaffar et al.** (55) | 2022 | 1 | partial | 1 | 1 | 1 | 1 | partial | 1 | 0 | Moderate |
| **Aqeel et al.** (56) | 2023 | 1 | partial | partial | 1 | 1 | partial | partial | 1 | 0 | High |
| **Bhatti et al.** (57) | 2023 | 1 | partial | partial | 1 | 1 | partial | partial | 1 | 0 | High |
| **Tariq et al.** (58) | 2023 | 1 | partial | 1 | 1 | 1 | 1 | partial | 1 | 0 | Moderate |
| **Saeed et al.**(59) | 2023 | 1 | partial | 1 | 1 | 1 | 1 | partial | 1 | 0 | Moderate |
| **Ahmed et al.** (60) | 2023 | partial | 0 | 1 | 1 | 1 | 1 | 1 | 1 | unclear | Moderate |
| **Rana et al.**(61) | 2024 | partial | 0 | 0 | 1 | 1 | 1 | partial | 1 | 0 | High |
| **Izhar et al .**(62) | 2025 | partial | 1 | 1 | 1 | 1 | 1 | 1 | 1 | unclear | Low |
| **Talat et al.**(63) | 2025 | 1 | 1 | 1 | 1 | 1 | 1 | 1 | 1 | 1 | Low |
| **Basharat et al.**(64) | 2025 | 0 | 0 | 1 | 1 | 1 | 1 | 1 | 1 | unclear | Moderate |
| **Mehmood et al.**(65) | 2025 | 1 | unclear | 1 | 1 | 1 | 1 | 1 | 1 | unclear | Low |
| **Jamal et al.** (66) | 2006 | partial | 0 | partial | 1 | partial | 1 | partial | 1 | 0 | High |
| **Wahab et al.** (67) | 2008 | 1 | 1 | partial | 1 | 1 | 1 | partial | 1 | 1 | Low |
| **Ghauri et al.** (68) | 2010 | 1 | partial | 1 | 1 | 1 | 1 | partial | 1 | 0 | Moderate |
| **Shaikh et al.** (69) | 2010 | 1 | 1 | 1 | 1 | 1 | 1 | 1 | 1 | 1 | Low |
| **Mahar et al.** (70) | 2010 | 1 | 1 | 1 | 1 | 1 | 1 | 1 | 1 | partial | Low |
| **Khanzada et al.** (71) | 2011 | 1 | 1 | 1 | 1 | 1 | 1 | 1 | 1 | 1 | Low |
| **Memon et al.** (72) | 2013 | 1 | 1 | 1 | 1 | 1 | 1 | 1 | 1 | 1 | Low |
| **Naveed et al.** (73) | 2014 | partial | 0 | 0 | 1 | 1 | 1 | unclear | 0 | unclear | High |
| **Alkhairy et al.** (74) | 2015 | partial | 0 | 1 | 1 | 1 | 1 | 1 | partial | unclear | Moderate |
| **Ishaq et al.** (75) | 2016 | 1 | partial | partial | 1 | 1 | 1 | partial | 1 | 0 | Moderate |
| **Nasir et al.** (76) | 2020 | partial | 0 | 1 | 1 | 1 | 1 | partial | 1 | 0 | Moderate |
| **Jokhio et al.** (77) | 2022 | 1 | 1 | 1 | 1 | 1 | 1 | 1 | 1 | 1 | Low |
| **Huma et al.** (78) | 2023 | 1 | partial | 1 | 1 | 1 | 1 | partial | 1 | 0 | Moderate |
