# Supplementary material for: The prevalence of diabetic retinopathy in type-2 diabetes in Pakistan: a systematic review and meta-analysis
Source: Front Clin Diabetes Healthc. 2026 Mar 30;7:1758759. doi: 10.3389/fcdhc.2026.1758759 (PMC13070823; doi:10.3389/fcdhc.2026.1758759)
Supplement: Supplementary file 5 [file Table5.docx]

**supplementary file 5**

**Sensitivity Analysis**

Study Events 95%-CI tau^2 tau I^2 95%-PI

Omitting Marwat et al. (26) 2012 0.3186 [0.2698; 0.3696] 0.0425 0.2062 98.5% [0.0314; 0.7245]

Omitting Saleem et al. (32) 2024 0.3211 [0.2704; 0.3741] 0.0458 0.2140 98.7% [0.0268; 0.7409]

Omitting Ahmed et al. (60) 2023 0.3217 [0.2706; 0.3750] 0.0464 0.2154 98.7% [0.0261; 0.7440]

Omitting Khan et al.(33) 2025 0.3227 [0.2711; 0.3766] 0.0473 0.2176 98.8% [0.0250; 0.7487]

Omitting Salman et al. (58) 2023 0.3232 [0.2713; 0.3773] 0.0478 0.2186 98.7% [0.0245; 0.7510]

Omitting Khan et al. (29) 2021 0.3239 [0.2717; 0.3783] 0.0483 0.2198 98.8% [0.0240; 0.7536]

Omitting Basharat et al.(64) 2025 0.3239 [0.2717; 0.3784] 0.0484 0.2199 98.7% [0.0239; 0.7540]

Omitting Talat et al.(63) 2025 0.3252 [0.2725; 0.3802] 0.0492 0.2218 98.8% [0.0231; 0.7584]

Omitting Sohail et al. (36) 2014 0.3254 [0.2726; 0.3804] 0.0493 0.2220 98.8% [0.0231; 0.7588]

Omitting Ghaffar et al. (55) 2022 0.3254 [0.2726; 0.3805] 0.0493 0.2221 98.7% [0.0230; 0.7592]

Omitting Mujtaba et al.(35) 2025 0.3256 [0.2728; 0.3807] 0.0494 0.2222 98.8% [0.0230; 0.7595]

Omitting Alkhairy et al. (73) 2015 0.3255 [0.2727; 0.3807] 0.0494 0.2222 98.8% [0.0229; 0.7595]

Omitting Qayyum et al. (22) 2010 0.3256 [0.2728; 0.3808] 0.0494 0.2223 98.6% [0.0229; 0.7597]

Omitting Jamil et al.(34) 2025 0.3263 [0.2733; 0.3816] 0.0497 0.2229 98.8% [0.0227; 0.7614]

Omitting Izhar et al .(62) 2025 0.3265 [0.2734; 0.3818] 0.0498 0.2231 98.8% [0.0227; 0.7618]

Omitting Safila et al. (72) 2014 0.3269 [0.2738; 0.3823] 0.0499 0.2234 98.8% [0.0227; 0.7628]

Omitting Rana et al.(61) 2024 0.3273 [0.2742; 0.3828] 0.0500 0.2236 98.8% [0.0228; 0.7634]

Omitting Shera et al. (19) 2004 0.3275 [0.2743; 0.3831] 0.0501 0.2239 98.8% [0.0226; 0.7641]

Omitting Ishaq et al. (74) 2016 0.3276 [0.2743; 0.3832] 0.0501 0.2238 98.8% [0.0226; 0.7641]

Omitting Hassan et al.(39) 2010 0.3278 [0.2745; 0.3834] 0.0502 0.2240 98.8% [0.0226; 0.7646]

Omitting Khanzada et al. (70) 2011 0.3279 [0.2746; 0.3835] 0.0502 0.2240 98.8% [0.0226; 0.7647]

Omitting Chachar et al. (54) 2022 0.3286 [0.2752; 0.3843] 0.0503 0.2242 98.8% [0.0227; 0.7656]

Omitting Saeed et al.(59)] 2023 0.3287 [0.2753; 0.3844] 0.0503 0.2242 98.8% [0.0227; 0.7659]

Omitting Farasat et al. (48) 2017 0.3292 [0.2758; 0.3849] 0.0503 0.2242 98.8% [0.0229; 0.7663]

Omitting Adnan et al. (42) 2014 0.3293 [0.2759; 0.3850] 0.0503 0.2242 98.8% [0.0230; 0.7662]

Omitting Aamir et al. (27) 2012 0.3294 [0.2759; 0.3851] 0.0503 0.2243 98.8% [0.0229; 0.7665]

Omitting Huma et al. (77) 2023 0.3301 [0.2766; 0.3858] 0.0502 0.2241 98.8% [0.0232; 0.7669]

Omitting Shahzad et al. (52) 2020 0.3302 [0.2767; 0.3859] 0.0502 0.2241 98.8% [0.0233; 0.7669]

Omitting Mahar et al. (69) 2010 0.3302 [0.2767; 0.3859] 0.0502 0.2241 98.8% [0.0233; 0.7670]

Omitting Jawa et al. (21) 2016 0.3303 [0.2768; 0.3860] 0.0502 0.2241 98.8% [0.0233; 0.7671]

Omitting Shaikh et al. (68) 2010 0.3305 [0.2771; 0.3862] 0.0502 0.2240 98.8% [0.0235; 0.7670]

Omitting Memon et al. (71) 2013 0.3306 [0.2771; 0.3863] 0.0502 0.2240 98.8% [0.0235; 0.7671]

Omitting Jokhio et al. (76) 2022 0.3308 [0.2773; 0.3865] 0.0501 0.2239 98.8% [0.0236; 0.7672]

Omitting Hussain et al. (41) 2013 0.3308 [0.2774; 0.3865] 0.0501 0.2239 98.8% [0.0237; 0.7671]

Omitting Junaid et al. (30) 2023 0.3310 [0.2776; 0.3867] 0.0500 0.2237 98.8% [0.0239; 0.7670]

Omitting Afghani et al. (38) 2007 0.3312 [0.2777; 0.3869] 0.0500 0.2237 98.7% [0.0239; 0.7672]

Omitting Mehmood et al.(64) 2025 0.3312 [0.2778; 0.3869] 0.0500 0.2237 98.8% [0.0239; 0.7671]

Omitting Saleem et al. (43) 2014 0.3312 [0.2778; 0.3869] 0.0500 0.2236 98.8% [0.0240; 0.7670]

Omitting Sajid et al (24) 2023 0.3315 [0.2781; 0.3871] 0.0499 0.2235 98.8% [0.0241; 0.7670]

Omitting Sardar et al. (51) 2019 0.3315 [0.2781; 0.3871] 0.0499 0.2235 98.8% [0.0241; 0.7670]

Omitting Ghauri et al. (67) 2010 0.3316 [0.2782; 0.3871] 0.0499 0.2234 98.8% [0.0243; 0.7668]

Omitting Hayat et al. (25) 2012 0.3321 [0.2788; 0.3876] 0.0497 0.2230 98.8% [0.0247; 0.7666]

Omitting Khalid et al. (45) 2015 0.3323 [0.2790; 0.3878] 0.0497 0.2228 98.8% [0.0248; 0.7667]

Omitting Manzoor et al. (49) 2018 0.3323 [0.2791; 0.3878] 0.0496 0.2227 98.8% [0.0250; 0.7665]

Omitting Mehreen et al. (50) 2018 0.3323 [0.2791; 0.3878] 0.0496 0.2227 98.8% [0.0250; 0.7665]

Omitting Khwaja et al. (28) 2019 0.3323 [0.2791; 0.3878] 0.0496 0.2227 98.8% [0.0250; 0.7665]

Omitting Aqeel et al. (56) 2023 0.3323 [0.2791; 0.3878] 0.0496 0.2227 98.8% [0.0250; 0.7665]

Omitting Nasir et al. (75) 2020 0.3324 [0.2792; 0.3879] 0.0496 0.2227 98.8% [0.0250; 0.7665]

Omitting Khan et al. (31) 2023 0.3324 [0.2792; 0.3879] 0.0496 0.2227 98.8% [0.0250; 0.7665]

Omitting Uddin et al. (37) 2018 0.3325 [0.2792; 0.3880] 0.0496 0.2226 98.8% [0.0250; 0.7665]

Omitting Wahab et al. (66) 2008 0.3325 [0.2793; 0.3879] 0.0496 0.2226 98.8% [0.0251; 0.7664]

Omitting Shaikh et al. (20) 2008 0.3326 [0.2794; 0.3881] 0.0495 0.2225 98.8% [0.0252; 0.7664]

Omitting Bhatti et al. (57) 2023 0.3326 [0.2794; 0.3880] 0.0495 0.2225 98.8% [0.0252; 0.7663]

Omitting Khan et al. (44) 2015 0.3327 [0.2795; 0.3881] 0.0494 0.2224 98.8% [0.0253; 0.7662]

Omitting Qamar et al. (46) 2016 0.3328 [0.2796; 0.3882] 0.0494 0.2223 98.8% [0.0253; 0.7662]

Omitting Riaz et al. (53) 2021 0.3328 [0.2796; 0.3882] 0.0494 0.2223 98.8% [0.0254; 0.7662]

Omitting Gardezi et al. (47) 2017 0.3327 [0.2796; 0.3881] 0.0494 0.2223 98.8% [0.0254; 0.7661]

Omitting Jamal et al. (65) 2006 0.3329 [0.2798; 0.3882] 0.0493 0.2221 98.8% [0.0256; 0.7660]

Omitting Hussain et al. (40) 2011 0.3337 [0.2808; 0.3889] 0.0489 0.2212 98.8% [0.0264; 0.7651]

Omitting Usman et al. (23) 2022 0.3339 [0.2811; 0.3890] 0.0488 0.2208 98.8% [0.0268; 0.7646]

**Random effects model 0.3292 [0.2767; 0.3840] 0.0494 0.2223 98.8% [0.0242; 0.7628]**
